# Valorization of Camel Milk Residue (CMR) into Hypoglycemic Peptides: An RSM-ANN Modeling Approach

**DOI:** 10.3390/foods14173086

**Published:** 2025-09-02

**Authors:** Han He, Yubin Cai, Yingying Ren, Shuyan Han, Liang Wang, Xuefeng Yin, Ayzohra Ablat, Abulimiti Yili, Ahmidin Wali, HajiAkber Aisa

**Affiliations:** 1 State Key Laboratory Basis of Xinjiang Indigenous Medicinal Plants Resource Utilization, Key Laboratory of Plant Resources and Chemistry in Arid Regions, Xinjiang Technical Institute of Physics and Chemistry, Chinese Academy of Sciences, Urumqi 830011, China; hehan23@mails.ucas.ac.cn (H.H.); caiyubin22@mails.ucas.ac.cn (Y.C.); hanshuyan23@mails.ucas.ac.cn (S.H.); ayizuohereabolaiti22@mails.ucas.ac.cn (A.A.);; 2 University of Chinese Academy of Sciences, Beijing 100049, China; 3 Key Laboratory of Xinjiang Phytomedicine Resource and Utilization, Pharmacy College of Shihezi University, Shihezi 832003, China; 15972192630@163.com; 4 College of Life Science and Technology, Xinjiang University, Urumqi 830000, China; wl1390593786@163.com (L.W.); yinxf591769130@163.com (X.Y.)

**Keywords:** camel milk residue, peptide, response surface methodology, artificial neural network, stability, *α*-glucosidase inhibition

## Abstract

This study valorized camel milk residue (CMR) via optimized bacterial fermentation to produce bioactive peptides with hypoglycemic potential. Screening of eleven bacterial strains identified four optimal starters. Artificial neural network (ANN) simulation significantly outperformed response surface methodology (RSM) in modeling and prediction, as evidenced by its superior performance in key statistical metrics, including R^2^, RMSE, and AAD(%), ultimately achieving a maximized yield of TCA-soluble nitrogen (TCA). Under optimized conditions, a TCA yield of 39.8% was achieved and experimentally validated. Ultrafiltration yielded a highly bioactive peptide fraction (<1 kDa), which exhibited significant inhibition of *α*-amylase (80.7%) and *α*-glucosidase (32.0%). The peptides exhibited high stability under various conditions, highlighting their industrial potential. This study explores the application of ANN-RSM optimization for the valorization of camel milk residue (CMR). Our findings provide a sustainable strategy for transforming CMR into a high-value anti-diabetic ingredient, which could contribute to extending the camel milk value chain.

## 1. Introduction

The global camel milk market is projected to reach USD 34.90 billion by 2034 [1], driving intensive production and industrial processing. Compared to cow milk, camel milk is richer in unsaturated fats (e.g., oleic acid), contains less cholesterol and sugar, and is a source of essential minerals [2]. More notably, it has garnered significant scientific interest for its anti-diabetic potential, attributed to bioactive peptides that may modulate insulin signaling. Evidence from both clinical and animal studies confirms its ability to significantly lower blood glucose, insulin demand, and improve lipid profiles [3,4].

However, this growth generates substantial waste, mainly spray-drying chamber residues (CMR), with annual yields reaching the ton-scale. Comprising fine particles and deposits (<80 μm), CMR represents not only a loss of valuable biomaterials but also a serious sustainability challenge due to its underutilization. Its valorization is therefore an urgent industrial priority. Bioconversion offers a promising strategy to transform such waste streams into high-value products, as demonstrated with by-products from other industries [5]. Analogously, CMR is an underutilized resource rich in bioactive compounds, yet its potential remains largely unexplored.

To address this gap, we propose an integrated approach using microbial fermentation to valorize CMR into hypoglycemic peptides. Given the complex, multi-strain bioprocess involved, this study employs a dual RSM-ANN modeling framework. While RSM statistically optimizes factor interactions, ANN captures nonlinear microbial synergies specific to CMR—a critical advantage for maximizing yield from variable waste streams. Furthermore, we emphasize not only peptide yield but also bioactivity and industrial applicability. The hypoglycemic effect is quantitatively assessed through *α*-amylase and *α*-glucosidase inhibition assays [6]. The stability of the peptide was assessed under rigorously simulated food processing conditions, encompassing a wide range of ionic strength (0.2–1.0 M NaCl), pH (2.0–7.4), temperature (4–60 °C), and exposure to organic solvents, including methanol, ethanol, and glycerol [7].

In summary, this work screens optimal microbial consortia for CMR fermentation, employs RSM-ANN to optimize the process, and functionally characterizes the resulting peptides. Our study provides a novel strategy for sustainable waste management in the camel milk industry and establishes a foundation for developing evidence-based anti-diabetic ingredients from CMR.

## 2. Materials and Methods

### 2.1. Materials

CMR was obtained from Xinjiang Zhongtuo Biotechnology Co., Ltd. (Yanchi Town, China). Dialysis bags and protein standards were purchased from Biosharp (Beijing, China). Enzyme labeling machines were purchased from Molecular Devices (San Jose, CA, USA), and centrifuges were obtained from Jiangsu Jinyi Instrument Technology Co., Ltd. (Changzhou, China). The microscope was from Motic (Xiamen, China). *α*-amylase and *α*-glucosidase were obtained from Beijing Aobo Star Biotechnology Co. (Beijing, China). Lipase (100,000 U/g) was purchased from Shandong Longke Keto Enzyme Preparation Co. (Linyi, China). Eleven commercial strains, *Bifidobacterium animalis* subsp. *lactis* CICC21712 (*B. lactis*), *Lacticaseibacillus case* CICC6114 (*L. case*), *Lactobacillus acidophilus* CICC6086 (*L. acidophilus*), *Lactococcus lactis* subsp. *lactis* CICC23196 (*L. lactis*), *Limosilactobacillus fermentum* CICC25124 (*L. fermentum*), *Limosilactobacillus reuteri* CICC6123 (*L. reuteri*), *Lactipalntibacillus plantarum* CICC25125 (*L. plantarum*), *Lactobacillus rhamnosus* CICC 6164 (*L. rha*), *Bifidobacterium thermophilum* CICC04950 (*B. thermophilum*), *Lacticaseibacillus paracasei* CICC6237 (*L. paracasei*), and *Pediococcus acidilactici* bio-097553, were sourced from the China Industrial Microbial Strains Preservation and Management Center (Beijing, China). The selection of these strains was based on the reference to Lv et al. [8].

### 2.2. Preparation of CMR Peptides

The method has been modified compared to that described in the previous study [9]. The dried CMR powder was sieved and dissolved in distilled water at a ratio of 1:25 (*w*/*v*) under vigorous stirring. After sterilization at 85 °C for 5 h, the mixture was chilled and held at ambient temperature. Then, lipase (100,000 U/g) was added at 0.1% *w*/*w* relative to the CMR powder weight. Enzymatic hydrolysis was performed at 50 °C for 6 h in a water bath, followed by enzyme inactivation at 85 °C for 10 min. The hydrolysate was cooled to room temperature. Following sterilization, a bacterial inoculum (1 × 10^7^ CFU/mL) was supplemented, with subsequent static fermentation at 37 °C for defined time [10]. Throughout the fermentation process, peptide levels were quantified every 2 h. Subsequent lyophilization (−80 °C) preceded storage at −40 °C.

### 2.3. Screening and Design of Experiments for RSM Modeling

According to the previous review [11], primary screening was conducted to determine the suitable ranges for key process parameters: fermentation time (X_1_: 2–18 h), fermentation temperature (X_2_: 33–41 °C), liquid-to-solid ratio (X_3_: 15–35:1), and bacterial density (X_4_: 5 × 10^6^–4 × 10^7^ CFU/mL) [12].

The experimental design employed a one-variable-at-a-time approach:

For X_1_ optimization (2–18 h), X_2_, X_3_, and X_4_ were fixed at 37 °C, 20:1, and 1 × 10^7^ CFU/mL, respectively.

For X_2_ optimization (33–41 °C), X_1_, X_3_, and X_4_ were fixed at 10 h, 20:1, and 1 × 10^7^ CFU/mL.

For X_3_ optimization (15–35:1), X_1_, X_2_, and X_4_ were fixed at 10 h, 37 °C, and 1 × 10^7^ CFU/mL.

For X_4_ optimization (5 × 10^6^–4 × 10^7^ CFU/mL), X_1_, X_2_, and X_3_ were fixed at 10 h, 37 °C and 20:1.

Preliminary experiments indicated that the response variable exhibited a unimodal trend: it increased initially and then decreased with increasing fermentation time (X_1_), fermentation temperature (X_2_), liquid-to-solid ratio (X_3_), and bacterial density (X_4_).

### 2.4. Ann Modeling

ANNs are machine learning algorithms inspired by biological neural systems [13]. They learn complex relationships between inputs and outputs through nonlinear mapping across multiple layers of neurons. An input layer, hidden layers, and an output layer form the standard ANN structure, among which backpropagation-based neural networks demonstrate the highest prevalence [14]. The unique value of ANNs in process optimization stems from their Universal Approximation Theorem, which theoretically guarantees their capability to approximate any continuous nonlinear function with arbitrary accuracy. Unlike RSM’s explicit mathematical model, ANNs function as “black-box” systems where internal relationships are not directly interpretable through parametric equations. Consequently, their optimization typically requires integration with intelligent algorithms such as Particle Swarm Optimization (PSO) or Genetic Algorithms (GA) to effectively navigate the parameter space. Empirical studies demonstrate that hybrid strategies like ANN-GA achieve superior prediction accuracy compared to pure RSM methodologies, as evidenced in applications like safflower seed oil extraction where ANN-GA significantly enhanced extraction yield prediction. AAD-based statistical analysis was applied to compare RSM and ANN model predictions [15], as given below:

(1) $$ADD(\%)=\;[\frac{\sum_{i=1}^{p}\left(\frac{\left|Y_{i,\exp}-Y_{i,\mathrm{cal}}\right|}{Y_{i,\exp}}\right)}{P}]\times 100$$

### 2.5. Peptide Content Analysis

Following Cotton’s protocol with little modification, 1 mL sample was mixed with 1 mL 15% (*w*/*w*) aqueous trichloroacetic acid (TCA) [16]. After 10 min equilibration, the mixture was centrifuged (4000 rpm, 10 min). Subsequently, 1 mL supernatant was mixed with 4 mL biuret reagent and vortexed. Absorbance at 540 nm determined hydrolysate peptide retention (C, mg/mL) via standard regression.

### 2.6. Kjeldahl Nitrogen Determination

After establishing optimal fermentation times per microbiome, samples were dried and analyzed for nitrogen concentration using the Kjeldahl method (GB 5009.5-2016) [17], employing a 6.25 protein conversion factor, and sample supernatants from multiple fermentation timepoints were centrifuged (4000 rpm, 10 min, 4 °C). Nitrogen concentration in the broth was quantified, with protein content derived therefrom [18].

### 2.7. Integrated Bacterial Proportion Quantification

Four dominant strains were screened from eleven candidates based on the results of the peptide content and hydrolysis degree for mixed fermentation. Uniform design experiments employed 4-strain inoculants with 11 factor-levels, 18 h fermentation, and peptide yield as the response. Quadratic polynomial stepwise regression (SPSS 20.0) generated predictive equations, with optimal inoculation ratios determined via Excel Solver 2023 [19,20].

### 2.8. MW Distribution Analysis

The main operating parameters were as follows:

Column: TSKgel 2000 SWXL (300 × 7.8 mm); mobile phase: acetonitrile/water/TFA (45:55:0.1, *v*/*v*); detection: UV 220 nm; flow rate: 0.5 mL/min; temperature: 30 °C.

Sample preparation: A100 mg sample was diluted to volume in 10 mL mobile phase within a volumetric flask then filtered (0.45 μm membrane).

Analysis: Fermentation time-series samples were analyzed under these conditions. Peptide profiles, molecular mass distributions, and ranges were derived via GPC software (1260 Infinity GPC/SEC System) processing [21].

### 2.9. Amino Acid Analysis

An 8 mg CMR was dissolved in 6 mol/L hydrochloric acid (HCl) containing 0.1% phenol and hydrolyzed at 110 °C for 24 h. Following acid hydrolysis, the solvent was completely removed by rotary evaporation under reduced pressure at 70 °C. The dried hydrolysate was reconstituted in 12 mL of ultrapure water [22].

For derivatization, 200 μL sample/amino acid standard was mixed with 100 μL 1 M triethylamine/acetonitrile and 100 μL 0.2 M PITC/acetonitrile, followed by 1 h reaction at room temperature (RT). Subsequently, 200 μL aqueous layer was collected, diluted with 800 μL H_2_O, and filtered (0.22 μm membrane).

The analysis employed HPLC fitted with a Diamonsil^®^ AAA column, with detection parameters maintained as specified in the prior methodology:

Eluent A: a 0.01 mol/L acetic acid–sodium acetate buffer (pH = 6.5 ± 0.05); eluent B: methanol/acetonitrile/water (20:60:20, *v*/*v*/*v*). A gradient elution program was employed. The chromatographic flow rate was sustained at 0.6 mL/min, with UV detection effected at 254 nm.

Constituent amino acids were characterized through retention time alignment against the reference mixed amino acid chromatogram. The relative percentage of each amino acid in the sample was determined by comparing the peak area ratios in the sample chromatogram. Tryptophan levels were determined via alkaline hydrolytic digestion per Landry and Delhaye [23].

### 2.10. Purification of CMR

Fractionation of the fermentation broth via centrifugal ultrafiltration tubes employing sequential molecular weight cut-offs (1, 3, and 10 kDa) generated discrete peptide fractions segregated by molecular mass. These fractionated peptides underwent lyophilization followed by cryopreservation at −80 °C to ensure molecular stability pending subsequent analytical procedures.

### 2.11. Determination of α-Amylase- and α-Glucosidase-Inhibitory Activities

Following the methodology described by Sadeghi et al. [24], with modifications, 30 μL aliquots of 2 mg/mL fractionated peptide solutions (varying MW) were combined with 30 μL PBS-buffered *α*-glucosidase (0.1 U/mL, pH 6.8). After adding 120 μL phosphate buffer, the mixture was incubated (37 °C, 25 min) in 96-well plates. Subsequently, 60 μL pNPG solution initiated enzymatic reactions, terminated by 70 μL sodium carbonate (0.8 M). Absorbance was recorded at 405 nm. Controls substituted *α*-glucosidase with equivalent PBS volume.

(2) Inhibition(
%)=1−A
1−A
2A3−A4 × 100%

A1: sample abs; A2: sample control abs; A3: blank abs; A4: blank control abs.

Following Huang et al.’s methodology with adaptations [25], 10 μL *α*-amylase (0.1 U/mL) was mixed with 10 μL fractionated samples in a 37 °C water bath. After 10 min incubation, 500 μL 0.9% soluble starch was added for 15 min extension. The reaction was terminated by boiling with 500 μL DNS reagent (8 min), cooled, and diluted with 4 mL distilled water. Absorbance of 250 μL aliquots in microplates was measured at 540 nm.

### 2.12. Inhibition Stability of CMR

Bioactive peptide stability fundamentally determines bioactivity. This investigation systematically assessed the impact of thermal stress, pH variance, metal ions, in vitro gastrointestinal simulation, organic solvents, and ionic strength on CMR peptide integrity.

#### 2.12.1. Impact of Ionic Strength on Stability of Various Fractions

A 2 mg/mL peptide solution was formulated, with NaCl gradients established through serial additions (0.2, 0.4, 0.6, 0.8, 1.0 mol/L) [26]. Post 2 h incubation, supernatants were harvested for dual analysis of peptide quantification and hypoglycemic activity.

#### 2.12.2. Impact of pH on Stability of Various Fractions

Peptides were solubilized in 0.1 M Na_2_HPO_4_-citrate buffers (pH 2–12) at 2 mg/mL [27]. pH was adjusted with 1 M HCl/NaOH, followed by 2 h equilibration at room temperature prior to quantifying peptide content and hypoglycemic activity.

#### 2.12.3. Impact of Temperature on Stability of Various Fractions

Fractionated peptide solutions (2 mg/mL) underwent aqueous bath incubation at 60 °C to 100 °C for 2 h [28]. Samples were subsequently quench-cooled to ambient temperature via ice-water bath immersion—a critical step to preserve structural integrity prior to the quantitative assessment of peptide levels and hypoglycemic bioactivity.

#### 2.12.4. Impact of Organic Solvents on Stability of Various Fractions

Fractionated peptide solutions (2 mg/mL) were individually blended with methanol, ethanol, and glycerol at graded concentrations (10%, 20%, 30%, 40%, 50%). Post 2 h agitation, peptide quantification and hypoglycemic activity assessment were conducted.

### 2.13. Peptide Characterization

To elucidate structure–function relationships, fractionated peptide populations spanning discrete molecular weight ranges underwent rigorous spectroscopic characterization. Fourier transform infrared spectroscopy (FT-IR) probed secondary structure motifs through amide I/II band analysis, while ultraviolet–visible spectroscopy (UV-vis) quantified aromatic residue interactions and charge-transfer complexes [29]. Employing standardized KBr pellet methodology under nitrogen-purged desiccation, visible-phase samples were precisely blended with anhydrous spectroscopic-grade KBr (1:100 sample-to-matrix ratio) in an agate mortar. Sequential grinding cycles achieved homogeneous micronization prior to hydraulic pressing into 13 mm translucent disks. FT-IR analysis implemented rigorous background subtraction with 64-scan averaging, followed by sample spectral acquisition at 4 cm^−1^ resolution across 4000–400 cm^−1^. For UV-vis characterization, samples were reconstituted in HPLC-grade water and analyzed using a UV spectrophotometer with 1.0 nm slit width. Full-spectrum scanning employed matched quartz cuvettes.

### 2.14. Statistical Analysis

Data are presented as mean ± SD (triplicate measurements). Statistical significance was determined by one-way ANOVA with Duncan’s post hoc test (*p* < 0.05). Modeling utilized Design Expert V13 and MATLAB R2024a, with model performance evaluated through the following equations:

(3) $$R^{2}\;=1-\frac{\sum_{i=1}^{n}{\left(y_{i}-Y_{i}\right)}^{2}}{\sum_{i=1}^{n}{\left(y_{i}-y\right)}^{2}}$$

(4) $$AAD(\%)=\left[\frac{\sum_{i=1}^{n}\left(\frac{\left|y_{i}-Y_{i}\right|}{Y_{i}}\right)}{n}\right]\times 100$$

(5) $$RMSE=\sqrt{\frac{1}{n}\sum_{i=1}^{n}{\left(y_{i}{-Y}_{i}\right)}^{2}}$$

## 3. Results and Discussion

### 3.1. Comparative Modeling of CMR Production Using Response Surface Methodology and Artificial Neural Networks

#### 3.1.1. Screening of Dominant Strains and Determination of Their Optimal Proportions

**Figure 1:**
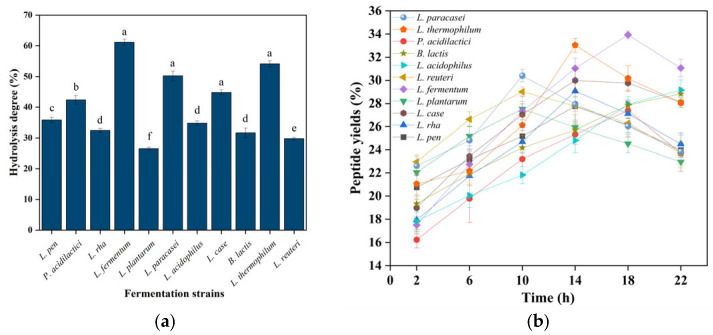
(**a**) Strain-specific hydrolysis degrees (%) in fermentation residues. (**b**) Temporal peptide yield (%) profiles during CMR fermentation. Mean ± SD, n = 3; *p* < 0.05. Different lowercase letters indicate significant differences.

Strain selection for camel milk fermentation requires consideration of unique functional characteristics. Mixed-culture fermentation demonstrates superior performance to monocultures due to microbial synergism [30]. From 11 candidate strains, dominant performers were selected for subsequent co-fermentation based on single-strain evaluation (Figure 1b). *L. fermentum*, *L. paracasei*, *L. casei*, and *L. thermophilus* demonstrated superior peptide production with optimal fermentation times of 8–18 h (Figure 1b). These strains exhibited characteristic biphasic growth kinetics, with peptide production peaking before declining, suggesting protease depletion or product inhibition. In contrast, *L. acidophilus* and *B. lactis* showed linear growth but minimal peptide yield (≤0.8 mg/mL). The observed performance differences likely reflect strain-specific protease secretion patterns and metabolic capabilities. Mixed-culture fermentation was employed to capitalize on synergistic interactions between selected strains, addressing the limitations of monoculture systems [31]. This approach enhances both peptide yield and diversity through complementary proteolytic activities targeting different cleavage sites in camel milk proteins.

#### 3.1.2. Measurement of Protein Hydrolysis

Nitrogen quantification of peak-yield fermentation residues from 11 strains verified CMR hydrolysis extent, with all broths exceeding 28% hydrolytic cleavage (Figure 1a). Ma et al. [12] conducted microbial fermentation of chickpea proteins, followed by post-fermentation nitrogen content assessment. This microbial consortium exhibits broad environmental adaptability, facilitating secondary proteolysis that enriches fermentation products with bioactive peptides. Hou et al. [32] demonstrated that fermentation significantly enhances proteolytic efficiency (*p* < 0.01), yielding bioactive-enriched protein hydrolysates with superior functional properties. Strain prioritization employed quantitative thresholds: peptide yield > 15 mg/mL and hydrolysis degree > 28%, identifying four dominant strains, namely, *L. fermentation*, *L. paracasei*, *L. casei*, and *L. thermophilus*.

#### 3.1.3. Homogeneous Design for Strain Ratio Optimization

Consortia fermentation demonstrates superior growth kinetics, enhancing product quality while suppressing off-flavor formation. Scholarly evidence [33] confirmed that mixed-culture systems exhibit enhanced proteolytic functionality, particularly in facilitating the release of bioactive hydrolysates during cheese maturation processes. Consequently, uniform design methodology (Table 1) implemented the peptide production rate as the response metric under 18 h fermentation to optimize strain ratios. A quadratic polynomial stepwise regression model was derived as follows:

(6) Y = −119.53 + 680.2 x_1_ + 626.4 x_3_ − 453 x_1_x_3_ + 28.5 x_1_x_4_ − 1426 x_1_^2^ − 920 x_3_^2^

The regression equation was R^2^ = 0.99. The final optimal mix of bacteria was noted to be *L. fermentation*, 16.4%; *L. thermophilus*, 28.0%; *L. paracasei*, 36.7%; and *L.casei*, 18.9%. The predicted peptide yield at this point was 39.62%. Validation under model-predicted optimum conditions yielded 39.04 ± 0.28% peptide from CMR fermentation, demonstrating high predictive accuracy and model validity.

#### 3.1.4. Results of RSM and Analysis of Variance

**Figure 2:**
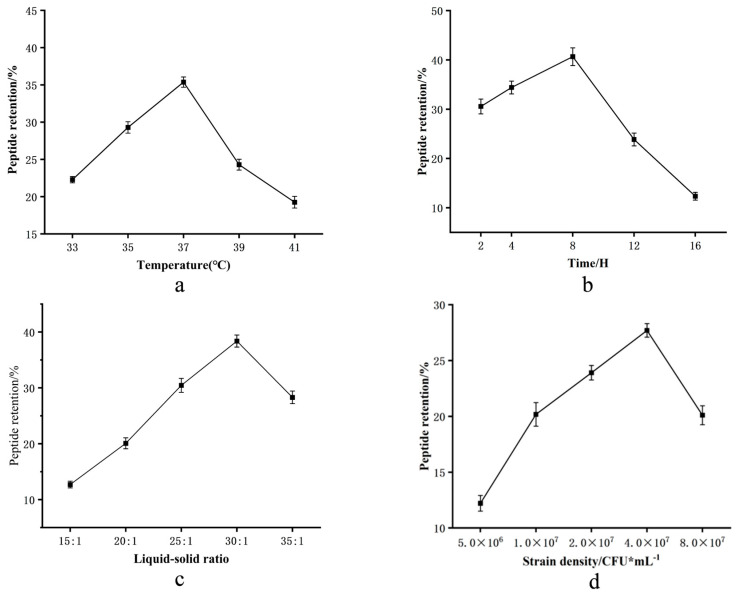

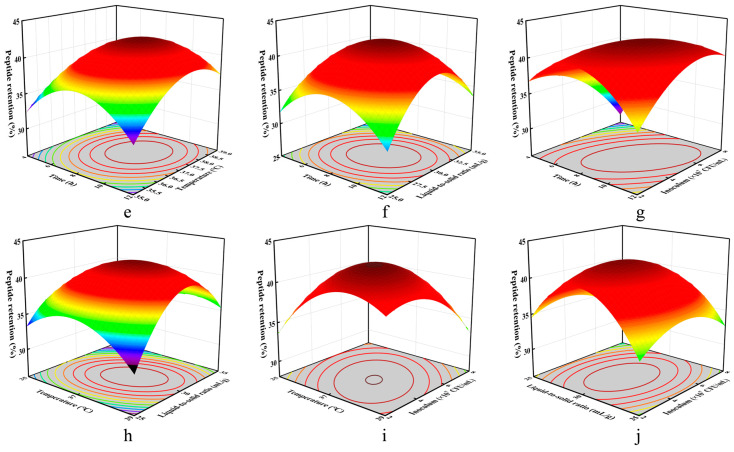
(**a**–**d**) Single-factor effects on CMR’s TCA: temperature, fermentation time, liquid–solid ratio, and inoculum. (**e**–**j**) Paired interactions: temperature × time; temperature × ratio; temperature × inoculum; time × ratio; time × inoculum; ratio × inoculum.

Microbial metabolism and product quality depend on fermentation conditions. We employed response surface methodology (RSM) to optimize conditions for TCA production, using TCA content as the response value. Preliminary single-factor experiments identified fermentation time (Table 2) (A), temperature (B), liquid-to-solid ratio (C), and inoculum amount (D) as key factors, determining their optimal ranges:

Time (A): TCA peaked at 39.8% after 8 h (Figure 2a).

Temperature (B): Maximum TCA yield (37.7%) occurred at 37 °C, within the expected mesophilic range (Figure 2b).

Ratio (C): A liquid-to-solid ratio of 30:1 yielded the highest TCA (39%) (Figure 2c).

Inoculum (D): TCA production reached 38% at 4 × 10^7^ CFU/mL (Figure 2d).

(7) Y = −2328.88741 + 6.06A + 115.61B + 11.84C + 13.65D + 0.014AB + 0.0044AC + 0.011AD + 0.031BD − 0.22CD − 0.41A^2^ − 1.56B^2^ − 0.21C^2^ − 0.47D^2^

**Table 2:** The peptide yields of CMR fermented by eleven separate strains.

Degree of Hydrolysis/%		
Source	*F*-Value	*p*-Value
Model	12.17	<0.0001 **
A-Fermentation time	1.09	0.0314 *
B-Temperature	2.14	0.0165 *
C-ratio	0.0865	0.0077 *
D-Inoculum amount	26.71	<0.0001 **
AB	0.0154	0.0903
AC	0.9244	0.0093 *
AD	0.0217	0.0088 *
BC	0.1193	0.0735
BD	2.36	0.1468
CD	0.2164	<0.0001 **
A^2^	83.99	<0.0001 **
B^2^	78.15	<0.0001 **
C^2^	57.53	<0.0001 **
D^2^	26.09	0.0002 *
Lack of fit	1.41	0.4161 ^ns^
R^2^	0.9241	
Adj.R^2^	0.9033	
Pred.R^2^	0.8421	
Adeq. Precision	10.6988	
C.V.%	1.2911	

* *p* < 0.05, ** *p* < 0.01, ^ns^ means not significant.

Analysis of variance (ANOVA) confirmed that the model was highly significant (*p* < 0.01) with a non-significant lack of fit (*p* > 0.05). The high regression coefficient (R^2^ = 0.9241) demonstrated the model’s effectiveness in simulating and predicting optimal CMR fermentation parameters for TCA production.

To elucidate significant synergistic effects among fermentation parameters on TCA, three-dimensional response surface plots were generated. As evidenced in Figure 2e–j, TCA content initially increased with the incremental elevation of individual factor values, plateaued at the maximum response points, and subsequently declined beyond these optima.

#### 3.1.5. ANN Modeling and Comparison with RSM

**Figure 3:**
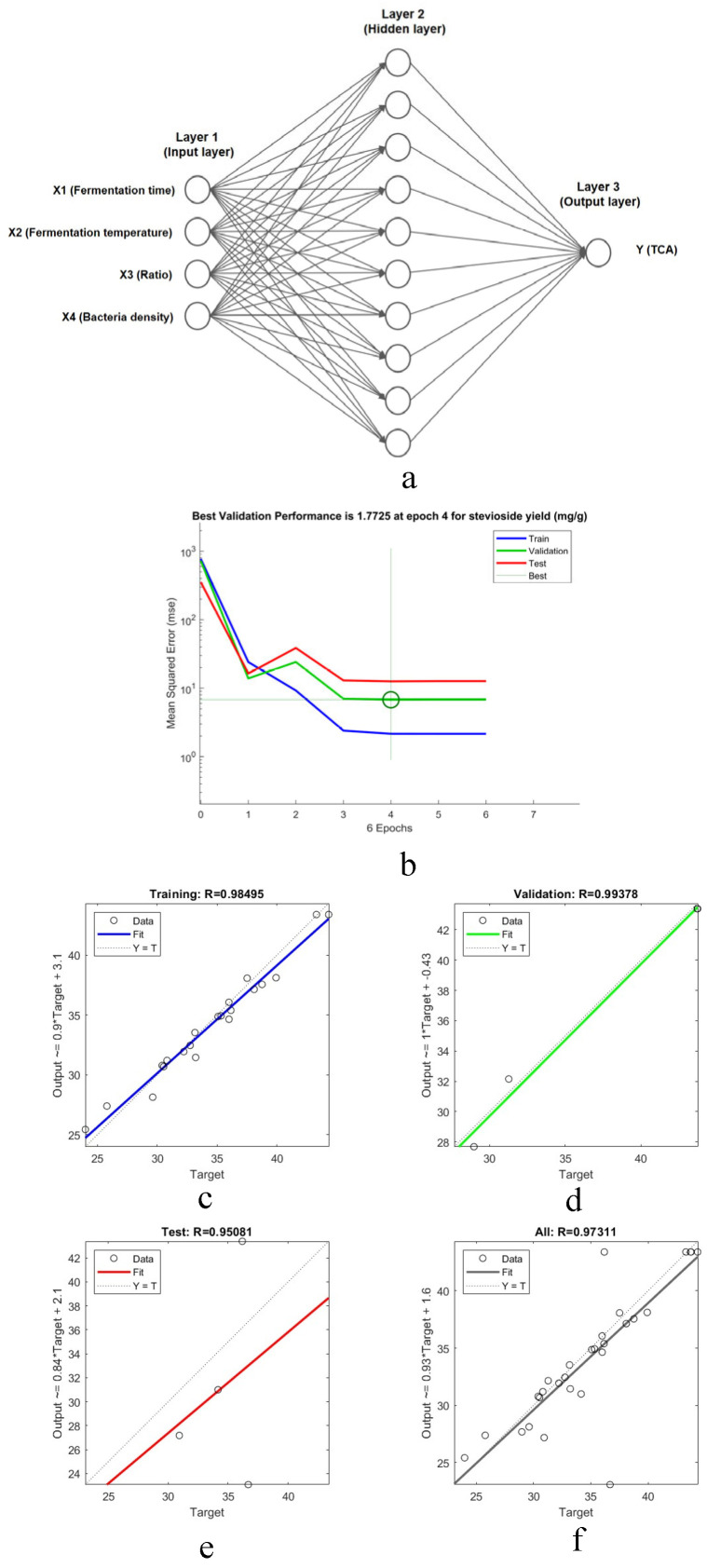
(**a**) Topology of the optimized ANN. (**b**) Epoch-dependent training profiles for TCA predicting subsets. (**c**–**f**) Regression analysis of CMR.

ANN model was developed to predict total TCA yield using the CMR process parameters (X1–X4) as inputs. The dataset was partitioned into training (70%, 20 samples), validation (15%, 5 samples), and testing (15%, 5 samples) sets. The Levenberg–Marquardt algorithm minimized mean square error to optimize weights and biases. The optimal architecture, determined as 3:10–20–10:1 (input: hidden1: hidden2: output neurons), is shown in Figure 3a.

Regression analysis (Figure 3b) yielded high correlation coefficients (R^2^ > 0.9241) across training, validation, and testing, demonstrating the model’s reliability. Predictive performance was compared with the RSM model (Figure 3c–f, Table 3). The ANN model exhibited superior accuracy, evidenced by a higher R^2^ (0.9469 vs. 0.9241), lower RMSE (5.582 vs. 5.676), and lower AAD (1.996 vs. 2.027), consistent with literature reports of ANN’s enhanced fitting and predictive capability. The CMR-ANN dual-model approach was selected owing to the complex nature of the camel milk residue (CMR), which contains a diverse array of peptides. Nonetheless, this study acknowledges the potential limitations of the model and the scalability challenges that require further investigation.

#### 3.1.6. Optimization and Validation

Response surface fitting predicted optimal conditions (duration, 9.033 h; temperature, 37.13 °C; liquid-to-solid ratio, 30.408:1; inoculum concentration, 6 × 10^7^ CFU/mL) yielding a modeled TCA content of 39.85%. For practical application, parameters were adjusted to the following: duration, 9 h; temperature, 37 °C; liquid-to-solid ratio, 30:1; and inoculum concentration, 6 × 10^7^ CFU/mL. Experimental verification under these adjusted conditions yielded a TCA content of 39.96 ± 0.13% (mean ± SD, n = 3). This close agreement between the experimental result and the model prediction (39.96% vs. 39.85%) demonstrates the accuracy and reliability of the optimized fermentation conditions derived via RSM (Table 4).

**Table 4:** Peptide MW profiling from enzymatic CMR hydrolysis.

CMR	Molecular Weight (%)			
	<1 KDa	1–3 KDa	3–10 KDa	>10 KDa
0 h	6.42	4.08	40.26	51.07
8 h	91.27	6.13	2.1	0.51
18 h	92.21	5.84	1.6	0.36

#### 3.1.7. Molecular Weight Distribution

Molecular weight distribution analysis revealed significant changes during fermentation. The proportion of peptides >10 kDa decreased markedly from 51.07% to 0.51% within the first 8 h, indicating extensive proteolytic degradation of macromolecular proteins. Concurrently, the 1–3 kDa fraction declined to 6.13% at 8 h and further to 5.84% by 18 h, reflecting the continuous hydrolysis of intermediate-sized peptides (3–10 kDa). Sustained microbial proteolytic activity resulted in the predominant accumulation (>90%) of low-molecular-weight peptides (<1 kDa) by the end of fermentation.

**Figure 4:**
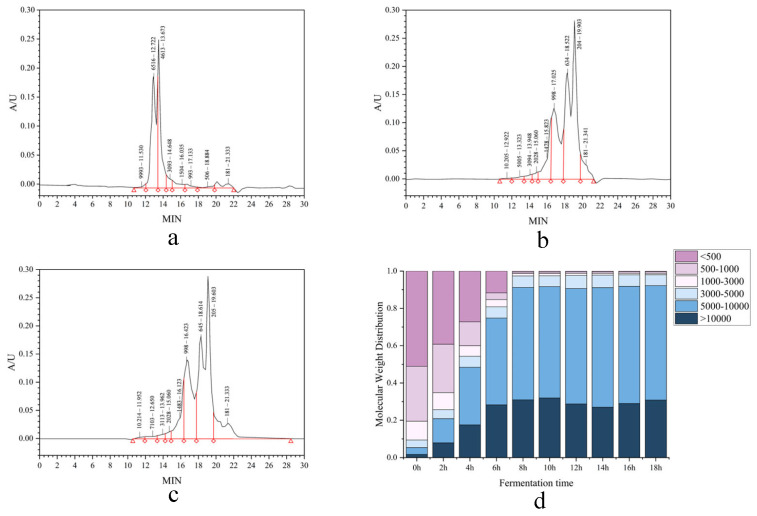
Temporal MW distribution profiling of CMR hydrolysates. (**a**–**c**) 0, 8, 18 h fermentation. (**d**) Comparative segmental distribution (<1, 1–3, 3–10, >10 kDa) across timepoints.

This pronounced shift towards low-molecular-weight fragments demonstrates the efficient bioconversion of macromolecular proteins into small peptides and amino acids. The profile suggests the enhanced bioavailability and metabolic stability of the resulting CMR peptide fractions. Studies by Zhao et al. [34] have demonstrated that low-molecular-weight peptides exhibit superior bioactivity. Therefore, this approach enables the establishment of a predictive framework linking specific hydrolysis parameters or purification steps to the resulting MW profile and, consequently, to the enhanced expression of desired biological activities. Ultimately, optimizing processes to enrich these low-MW fractions presents a strategic pathway for developing highly efficacious functional ingredients and nutraceuticals (Figure 4).

#### 3.1.8. Amino Acids Content

Total amino acid content exhibited biphasic kinetics, peaking at 1.932 ± 0.045 mg/mL by 8 h before declining to 1.786 ± 0.038 mg/mL at 18 h.

This reduction likely reflects the microbial assimilation of amino acids, minerals, and essential cofactors. Concurrently, maximal concentrations of essential amino acids (0.7904 mg/mL) and hydrophobic amino acids (0.8482 mg/mL) were achieved at 8 h.

The concurrent peak in bioactive amino acids and total content at 8 h establishes this duration as optimal for CMR fermentation, maximizing the yield of value-enhancing components. Therefore, an 8 h fermentation period is established as the optimal duration for CMR processing, effectively maximizing the liberation and concentration of bioactive amino acids with enhanced nutraceutical potential.

### 3.2. The α-Amylase and α-Glycosidase Inhibition Rate of Various Fractions

**Figure 5:**
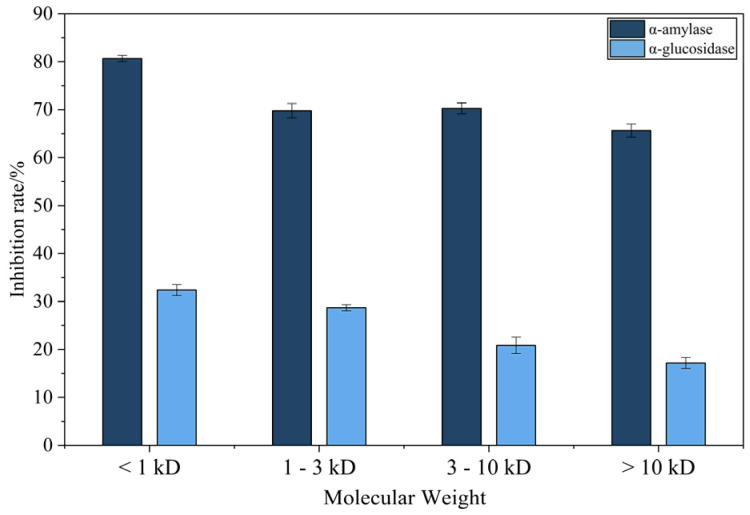
*α*-amylase and *α*-glycosidase inhibition rate by CMR fractions (2.0 mg/mL). Mean ± SD, n = 3 (*p* ≤ 0.05).

Ultrafiltration significantly enhanced the *α*-amylase- and *α*-glucosidase-inhibitory activities of fermented CMR (Figure 5). Among four molecular weight fractions (<1 kDa, 1–3 kDa, 3–10 kDa, >10 kDa) tested at 2.0 mg/mL, the <1 kDa fraction exhibited maximal inhibition (*α*-amylase: 80.66 ± 0.65%; *α*-glucosidase: 32.00 ± 1.15%), significantly surpassing the >10 kDa fraction (*p* < 0.05). No significant differences were observed between the 1–3 kDa or 3–10 kDa fractions and the <1 kDa fraction.

The superior bioactivity of the <1 kDa peptides aligns with reports on antihyperglycemic peptides (e.g., 43.82% inhibition by Antarctic krill hydrolysates [35]), suggesting that this fraction harbors abundant bioactive peptides responsible for potent enzyme inhibition (Table 5).

**Table 5:** Amino acid composition of CMR by different fermentation times.

Name/(mg/mL)	0 h	8 h	18 h
Asp	0.1735	0.1522	0.1356
Glu	0.1559	0.1799	0.1703
Gly	0.1141	0.0829	0.0854
His	0.0363	0.4042	0.3431
Arg	0.0542	0.0234	0.0147
Thr *	0.0573	0.0677	0.0626
Ala #	0.1520	0.2123	0.1876
Pro #	0.0670	0.1667	0.1743
Tyr	0.0423	0.0588	0.0356
Val *#	0.0970	0.1243	0.1277
Met *#	0.0472	0.1202	0.1034
Cys	0.0039	0.0694	0.0589
Ile *#	0.0336	0.0469	0.0415
Leu *#	0.0838	0.1207	0.1034
Phe *#	0.0332	0.0571	0.0329
Lys *	0.2043	0.2535	0.2459
Total amino acids	1.356	2.140	1.923

Note: * denotes essential amino acids; # denotes hydrophobic amino acids.

### 3.3. Inhibition Stability

#### 3.3.1. Ionic Strength-Dependent Stability Profiling of Fractionated CMR

**Figure 6:**
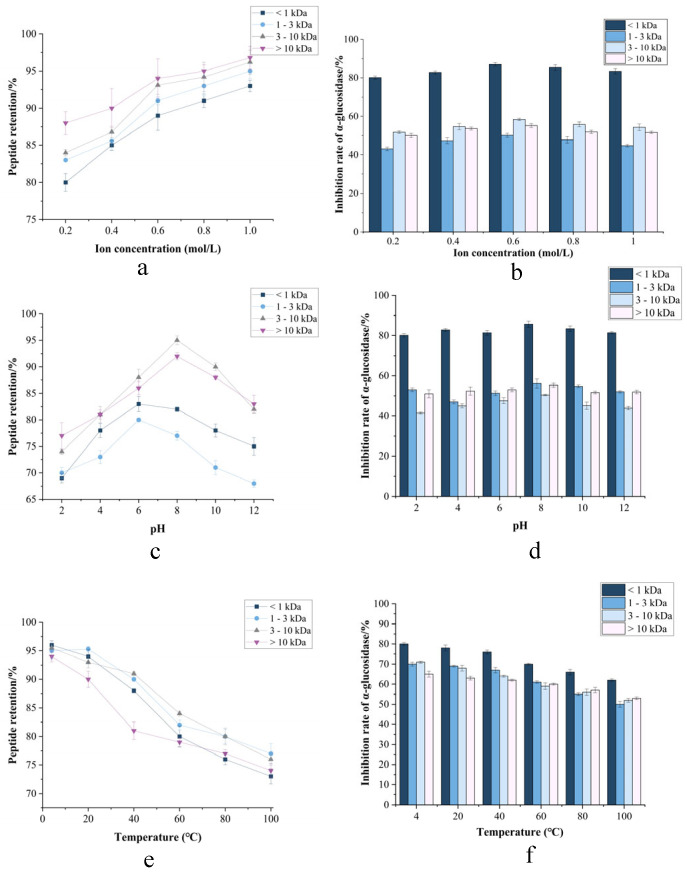
CMR stability under parametric modulation. (**a**,**c**,**e**) Peptide retention vs. ionic strength, pH, and temperature. (**b**,**d**,**f**) *α*-amylase and *α*-glucosidase inhibition profiles.

Ionic strength effects (Figure 6a) demonstrated remarkable peptide stability, with >80% retention across all molecular weight fractions (<1, 1–3, 3–10, >10 kDa) under NaCl concentrations up to 1.0 M. In contrast, the inhibitory activity of *α*-amylase and *α*-glycosidase in response to ionic strength varied significantly across these molecular weight fractions, generally exhibiting an initial increase followed by a decrease. Notably, the <1 kDa fraction displayed significantly higher inhibitory activity compared to the other fractions. This observed pattern aligns with findings reported by Mu et al. This insensitivity to ionic perturbation suggests that charge-shielding mechanisms effectively counteract salting-out effects, attributable to intrinsically low surface hydrophobicity of fermented peptides [36]. And the excellent ionic stability is attributed to the strong surface charge of the CMR, which provides electrostatic repulsion that prevents particle aggregation [37]. The stability of peptides under conditions of up to 1.0 M NaCl suggests their potential applications in fields such as food, health supplements, and pharmaceutical products, particularly in high-salt environments or for products requiring high stability.

#### 3.3.2. pH-Dependent Stability Profiling of Fractionated CMR

Particle stability must be assessed across pH 2.0–7.4, simulating gastric (pH ~2) to intestinal (pH 6.8–7.4) physiological transitions [38]. pH tolerance (Figure 6b) revealed unparalleled resilience: peptide retention exceeded 70% with >82% peptide retention throughout pH 2.0–10.0. This demonstrated excellent stability, as evidenced by its sustained inhibitory activity against *α*-amylase and *α*-glucosidase. Such extensive acid–alkali stability suggested that CMR can withstand the highly acidic gastric environment, indicating its potential application in gastric-targeted delivery [39].

#### 3.3.3. Temperature-Dependent Stability Profiling of Fractionated CMR

Thermal stability profiles (Figure 6c) exhibited critical molecular weight-dependent behavior. While all fractions maintained >85% retention at 4–60 °C, significant degradation occurred above 60 °C. The 3–10 kDa fraction showed pronounced susceptibility (23.7% activity loss at 80 °C; *p* < 0.01), whereas <1 kDa peptides demonstrated exceptional thermostability, retaining 82.58% integrity at 100 °C—comparable to Arthrospira platensis bioactive peptides [40]. Circular dichroism confirmed that thermal denaturation was driven by *α*-helix unfolding and *β*-sheet aggregation. Due to the inherently low molecular weight of the peptides inhibiting *α*-amylase and *α*-glucosidase, heating exerts minimal impact on their structure and stability. While low-MW peptides demonstrate superior thermal resistance, CMR bioactivity preservation mandates strict avoidance of elevated temperatures throughout storage, processing, and preparation due to irreversible structural denaturation [41]. Successful industrial application hinges on implementing gentle, low-temperature processing technologies throughout the entire production chain to leverage the full spectrum of bioactivities from all molecular weight fractions. This thermal liability, if managed correctly, translates into a high-value ingredient for premium health and wellness markets.

**Figure 7:**
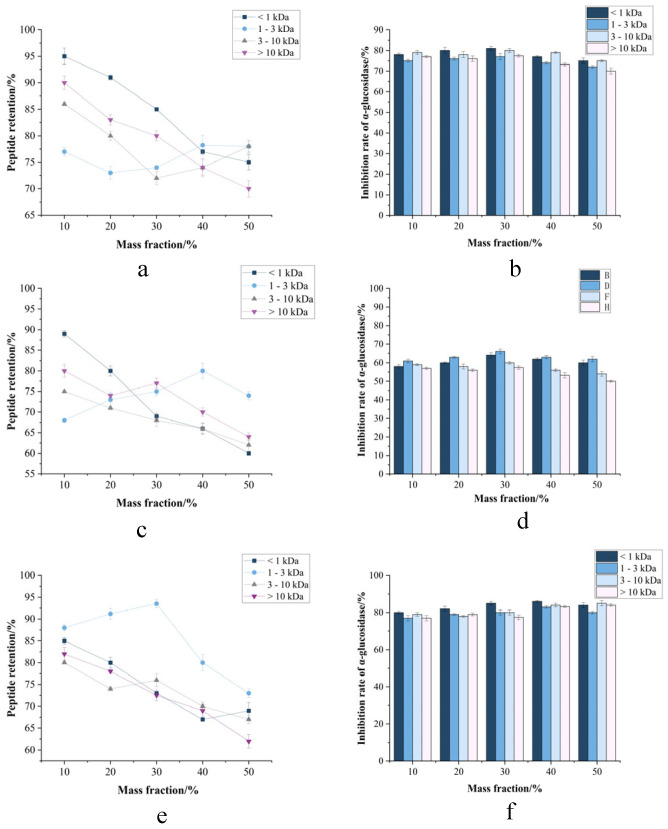
(**a**,**c**,**e**) Peptide retention vs. methanol/ethanol/propanol mass fractions. (**b**,**d**,**f**) *α*-amylase and *α*-glucosidase inhibition profiles.

#### 3.3.4. Organic Solvents-Dependent Stability Profiling of Fractionated CMR

Methanol and ethanol are ubiquitously employed in laboratory-scale extraction, purification, and analytical processes, such as serving as mobile phases in HPLC. Glycerol is a common additive in cosmetic formulations, where it functions as a humectant, solvent, or preservative. Although acetone is also a frequently used solvent, its application is predominantly restricted to specific lipid extraction processes. Therefore, methanol, ethanol, and acetone were selected for this investigation based on their prevalence in laboratory practices and relevance to extraction processes.

Organic solvent exposure (Figure 6d–f) induced concentration-dependent degradation, with critical thresholds at >30% (methanol), >25% (ethanol), and >40% glycerol (*v*/*v*). Maximum retention losses reached 62.3% (50% methanol). Notably, transient accumulation of 1–3 kDa peptides at low solvent concentrations (<15% *v*/*v*) occurred through reduced dielectric constant-mediated hydrophobic collapse, temporarily stabilizing intermediates via proteolytic shielding [42]. When the concentration of organic solvents exceeded 30%, a significant decrease in the inhibition rates against *α*-amylase and *α*-glucosidase was observed. This reduction is likely attributable to protein dehydration and aggregation, which leads to the burial of functionally critical hydrophobic and aromatic amino acids within hydrophilic residues. Consequently, CMR processing chains must minimize contact with organic solvents (e.g., methanol, ethanol, glycerol) during production, storage, and logistics [43]. Hence, for extraction or purification steps requiring organic solvents, the focus should be on using the lowest feasible concentration and shortest exposure time to leverage the stabilization window observed in the study, thereby minimizing degradation. Based on its organic solvent sensitivity, this CMR peptide is ideally suited for clean-label food, beverage, and nutraceutical applications that utilize solvent-free extraction and processing technologies, aligning with consumer demand for natural ingredients Figure 7.

### 3.4. Structural Characterization of Peptides

#### 3.4.1. UV

**Figure 8:**
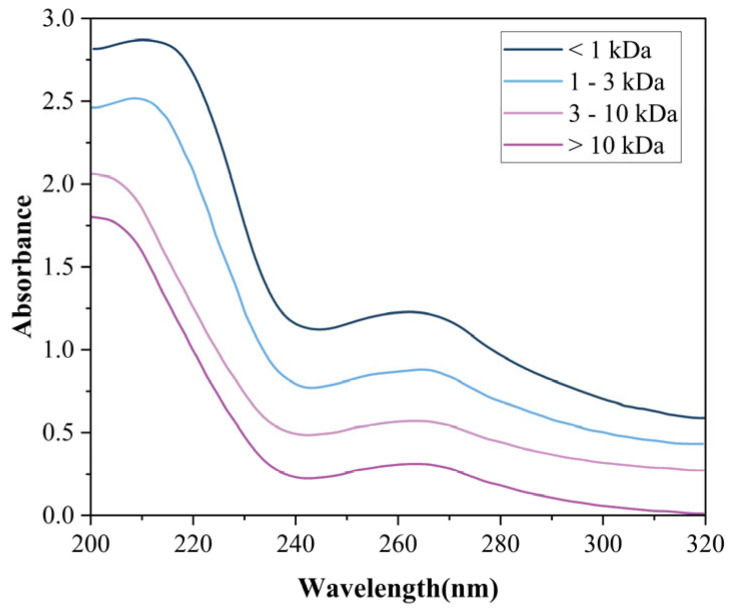
UV profiles of four MW-fractionated components.

UV-Vis spectra (Figure 8) revealed molecular weight-dependent absorption maxima at 214 nm (>3 kDa), 212 nm (1–3 kDa), and dual peaks at 209/202 nm (<1 kDa), characteristic of π→π* transitions in tryptophan/tyrosine residues [44]. Complementary absorptions at 210 nm (peptide backbone n→π* transitions) and 263 nm (aromatic amino acids) confirm conserved photophysical properties in plant-derived bioactive peptides, consistent with tomato peptide profiles reported by Hong et al. [45].

#### 3.4.2. FT-IR

**Figure 9:**
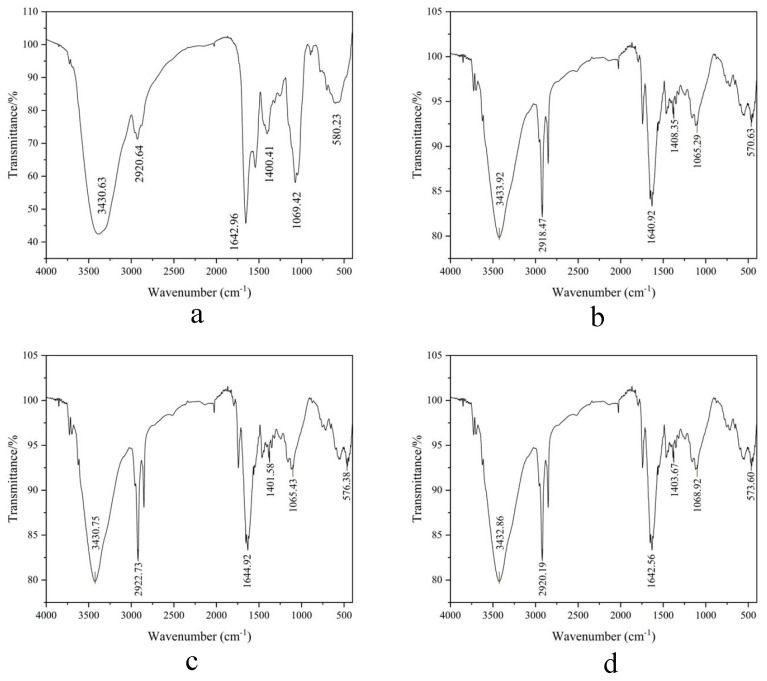
(**a**–**d**) FT-IR spectra profiles of four MW-fractionated components.

FT-IR analysis (Figure 9a–d) identified critical structural motifs: N-H stretching vibrations (3430–3434 cm^−1^), C-H bonds (2920 cm^−1^), and amide I C=O stretching (1640 cm^−1^). The dominant amide I band at 1600–1640 cm^−1^ signifies *β*-sheet-rich architectures [29], while minor *α*-helical signatures (1650–1660 cm^−1^) [28] indicate compact conformations stabilized by hydrogen-bonding networks. Minimal wavenumber shifts (<2 cm^−1^) in amide III (1400 cm^−1^, C-N stretching) verify structural integrity preservation throughout purification. These observations are consistent with the hypothesis that *β*-sheet domains may contribute to the observed functional stability [46].

## 4. Conclusions

This study established a protocol to identify 4 optimal bacterial strains from 11 candidates for camel milk residue (CMR) fermentation, a previously underexplored resource. Critically, we demonstrate for the first time the superior suitability of ANN over RSM for modeling CMR peptide fermentation, a key methodological innovation for precision bioprocessing. The resulting peptides, characterized as predominantly low-molecular-weight compounds with unique amino acid profiles, exhibited exceptional dual-enzyme inhibitory activity (*α*-amylase: 80.66 ± 0.65%; *α*-glucosidase: 32.00 ± 1.15%), significantly outperforming conventional dairy hydrolysates. Our pioneering integrated stability assessment, combining multifactorial stress tests (pH/ionic strength/temperature/solvents) with spectroscopic validation (UV/FT-IR), provides an unprecedented mechanistic framework for industrial process design and storage optimization. These findings collectively establish CMR-derived peptides as potent, multifunctional ingredients for glycemic management.

## Figures and Tables

**Table 1:** The peptide yields of CMR fermented by four separate strains.

No.	*L. casei* X1 (%)	*L. fermentum* X2 (%)	*L. thermophilus* X3 (%)	*L. paracasei* X4 (%)
1	15	17	20	29
2	16	18	21	30
3	17	19	22	31
4	18	20	23	32
5	19	21	24	33
6	20	22	25	34
7	21	23	26	35
8	22	24	27	36
9	23	25	28	37
10	24	26	29	38

**Table 3:** Predictive performance benchmarking: ANN versus RSM models.

Parameters	RSM	ANN
R^2^	0.9241	0.9469
RMSE	5.582	5.676
AAD(%)	2.027	1.996

## Data Availability

The original contributions presented in this study are included in the article. Further inquiries can be directed to the corresponding author.
